# Wnt/β-Catenin Signaling as a Molecular Target by Pathogenic Bacteria

**DOI:** 10.3389/fimmu.2019.02135

**Published:** 2019-09-27

**Authors:** Octavio Silva-García, Juan J. Valdez-Alarcón, Víctor M. Baizabal-Aguirre

**Affiliations:** ^1^Departamento de Química, Universidad del Papaloapan, Oaxaca, Mexico; ^2^Centro Multidisciplinario de Estudios en Biotecnología, Facultad de Medicina Veterinaria y Zootecnia, Universidad Michoacana de San Nicolás de Hidalgo, Morelia, Mexico

**Keywords:** bacteria, Wnt, β-catenin, infectious disease, signaling transduction, frizzled

## Abstract

The Wnt/β-catenin signaling pathway is crucial to regulate cell proliferation and polarity, cell determination, and tissue homeostasis. The activation of Wnt/β-catenin signaling is based on the interaction between Wnt glycoproteins and seven transmembrane receptors—Frizzled (Fzd). This binding promotes recruitment of the scaffolding protein Disheveled (Dvl), which results in the phosphorylation of the co-receptor LRP5/6. The resultant molecular complex Wnt–Fzd–LRP5/6-Dvl forms a structural region for Axin interaction that disrupts Axin-mediated phosphorylation/degradation of the transcriptional co-activator β-catenin, thereby allowing it to stabilize and accumulate in the nucleus where it activates the expression of Wnt-dependent genes. Due to the prominent physiological function, the Wnt/β-catenin signaling must be strictly controlled because its dysregulation, which is caused by different stimuli, may lead to alterations in cell proliferation, apoptosis, and inflammation-associated cancer. The virulence factors from pathogenic bacteria such as *Salmonella enterica* sv Typhimurium, *Helicobacter pylori, Mycobacterium tuberculosis, Pseudomonas aeruginosa, Citrobacter rodentium, Clostridium difficile, Bacteroides fragilis, Escherichia coli, Haemophilus parasuis, Lawsonia intracellularis, Shigella dysenteriae*, and *Staphylococcus epidermidis* employ a variety of molecular strategies to alter the appropriate functioning of diverse signaling pathways. Among these, Wnt/β-catenin has recently emerged as an important target of several virulence factors produced by bacteria. The mechanisms used by these factors to interfere with the activity of Wnt/β-catenin is diverse and include the repression of Wnt inhibitors' expression by the epigenetic modification of histones, blocking Wnt–Fzd ligand binding, activation or inhibition of β-catenin nuclear translocation, down- or up-regulation of Wnt family members, and inhibition of Axin-1 expression that promotes β-catenin activity. Such a variety of mechanisms illustrate an evolutionary co-adaptation of eukaryotic molecular signaling to a battery of soluble or structural components synthesized by pathogenic bacteria. This review gathers the recent efforts to elucidate the mechanistic details through which bacterial virulence factors modulate Wnt/β-catenin signaling and its physiological consequences concerning the inflammatory response and cancer.

## Introduction

**Figure 1:**
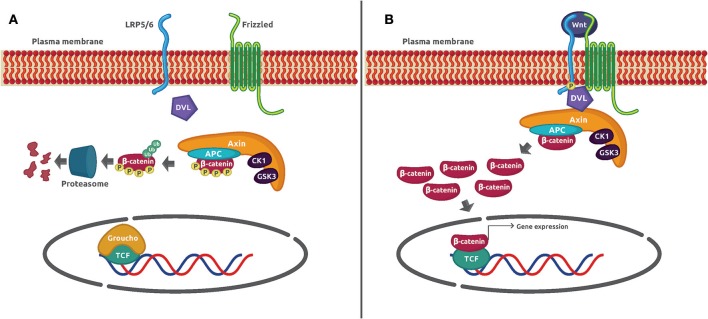
Wnt/β-catenin signaling mechanism. **(A)** In unstimulated cells, β-catenin forms a macromolecular complex with Axin, APC, CK1α, and GSK3 (*destruction complex*). Phosphorylation by CK1α and GSK3 prepares β-catenin to be ubiquitinated and degraded in the proteasome 26S. **(B)** The interaction of Wnt glycoprotein ligands with Frizzled receptors and LRP5/6 co-receptors recruits DVL to the plasma membrane. Then, GSK3 phosphorylates LRP5/6 and β-catenin is no longer phosphorylated and ubiquitinated because the *destruction complex* disassembles. The β-catenin accumulated in the cytoplasm is translocated to the nucleus where it displaces the co-repressor Groucho and interacts with TCF to activate gene expression.

The Wnt/β-catenin signaling pathway is an evolutionarily conserved mechanism that plays a preeminent role in maintaining cellular homeostasis. It regulates embryo development, cell proliferation and differentiation, apoptosis, and inflammation-associated cancer (1). In basal conditions, the protein transcriptional co-activator β-catenin is constantly phosphorylated by casein kinase 1α (CK1α) and glycogen synthase kinase 3β (GSK3β) in a process mediated by the Axin complex (also known as *destruction complex*) constituted by the scaffolding protein Axin, the tumor suppressor gene product adenomatous poliposis coli (APC), CK1α, and GSK3β. Such phosphorylation adds a chemical label to β-catenin that is recognized and polybuquitinated by the Skp1-Cdc53-F-Box E3 ubiquitin ligase complex (SCF^β−TRCP^) and degraded by the proteasome 26S. The activation of the canonical pathway is dependent on the binding of Wnt glycoprotein ligands to Frizzled (Fzd) receptors and its co-receptor, low-density lipoprotein receptor-related protein 5/6 (LRP5/6) on the plasma membrane. Several molecular events take place after the formation of the ternary complex Wnt–Fzd–LRP5/6. One of them is the translocation of the scaffolding protein disheveled (Dvl) to the complex, and the phosphorylation of LRP5/6 followed by the recruitment of the *destruction complex* to the receptor. The consequence of this series of protein–protein interactions is the inhibition of Axin-mediated β-catenin phosphorylation, which leads to β-catenin stabilization, cytoplasmic accumulation, and nuclear translocation. Once in the nucleus, β-catenin displaces the corepressor Groucho and binds to the DNA-bound transcription factors T-cell factor/lymphoid enhancer-binding factor (TCF/LEF) to activate Wnt-dependent gene expression (Figure 1).

The inflammatory response (IR) is one of the main defense mechanisms of the innate immune system that protects us against physical, chemical, or biological aggressions (2). The activation of different signaling pathways plays a fundamental role to promote, in the first place, or control, in later stages, the IR. Some of the most relevant transcription factors activated by these signaling mechanisms are the nuclear factor κB (NF-κB), the nuclear factor erythroid 2-related factor 2 (Nrf2), and the hypoxia-inducible factor 1α (HIF-1α) (3, 4). Interestingly, pro-inflammatory and anti-inflammatory roles have also been recently assigned to the Wnt/β-catenin signaling in different tissues stimulated with Wnt glycoproteins or infected by pathogenic bacteria. For example, the pro-inflammatory function was documented in pre-adipocytes and microglia cells stimulated with Wnt1 and Wnt3a, respectively (5, 6).

This review highlights new findings that strongly support the regulatory role of Wnt/β-catenin signaling components in the bacterial-induced IR and cancer. Each section contains concise but detailed descriptions on how pathogenic bacteria activate or inhibit the Wnt/β-catenin signaling and what physiological alterations they may cause. We have chosen to present pathogenic bacteria according to their impact on human health and the number of reports cited. Table 1 shows the most important effects on Wnt/β-catenin of each pathogenic bacteria discussed in text. It is our hope that discussing the activation or inhibition mechanisms of pathogenic bacteria on Wnt/β-catenin signaling will incentivize researchers to explore these molecular processes and propose new therapeutic targets.

**Table 1:** Effects of bacterial virulence factors on Wnt/β-catenin signaling pathway.

**Bacterium**	**Bacterial effector**	**Wnt/β-catenin pathway effect**	**Pathway component affected**	**References**
*Clostridium difficile*	Toxin B	↓ Reporter activity	↓FZD 1, 2, and 7 function	(7)
	Toxin A	↓ c-Myc expression ↓ Reporter activity	↓β-catenin stability	(8)
*Citrobacter rodentium*	Unknown	↑ c-Myc and Mmp7 expression	↓ WIF1 expression ↑β-catenin stability ↑ R-Spondin 2	(9) (10)
*Pseudomonas aeruginosa*	LecB	↓ c-Myc and cyclin D-1 expression	↓β-catenin stability	(11)
*Bacteroides fragilis*	Toxin	↑ c-Myc expression ↑ TCF reporter activity	↑β-catenin stability	(12)
*S. enterica typhimurium*	AvrA	↑ TCF reporter activity	↑Wnt2, Wnt3, Wnt6, Wnt9a, and Wnt11 ↑β-catenin stability	(13)
*Helicobacter pylori*	CagA	↑ c-Myc expression ↑ TCF reporter activity	↑β-catenin stability ↑ FZD7 expression ↓ WIF1 expression ↓ SFRP expression ↓ DKK expression ↓ Runx3/TCF4 dimer formation	(14–18)
*Mycobacterium tuberculosis*	Unknown	↑ c-Myc expression ↑ TCF reporter activity	↑ Wnt5a ↑FZD1 ↑β-catenin stability	(19)
*Escherichia coli*	LPS	↓ TAZ / WWTR1 expression	↓β-catenin	(20)
*Haemophilus parasuis*	Virulent strain	↓β-catenin–E-cadherin interaction	↑β-catenin nuclear translocation	(21)
*Shigella dysenteriae*	Virulent strain	↑ IL-8 ↑ TNFα	↑ phospho-β-catenin ↑ phospho-GSK3β	(22)
*Lawsonia intracellularis*	Virulent strain	↓ MUC2 ↑ Ki67 ↑Caspase-3	↑β-catenin	(23)
*Staphylococcus epidermidis*	LP78	↓ TNFα ↓ IL-6	↑ phospho-β-catenin	(24)

## *Salmonella enterica* sv Typhimurium: a Chronic Gastroenteritis That May Become Cancer

*Salmonella enterica* is one of the major public health issues in third- and first-world countries. According to statistical data in the United States, the number of illnesses caused by this enteric bacterium every year is about 1.2 million, with 23,000 hospitalizations and 450 deaths (25). Recent *S. enterica* outbreaks, linked to the consumption of a variety of foods such as tahini, raw chicken, and ground beef and having hedgehogs as pets, have been well-documented (25). Symptoms of acute *S. enterica* infection include fever and gastroenteritis; however, if *S. enterica* colonization becomes chronic, it may lead to other gastrointestinal disorders such as chronic inflammation and cancer. To deliver soluble protein virulence factors in the cytoplasm of host cells, this enteric bacterium is equipped with a type three secretion system (TTSS) (26), which is a nanostructure composed of a hollow needle that serves as a channel to inject the pathogenic virulence factors (called effectors). These *S. enterica* effectors can take on the role of eukaryotic molecules, changing the proper function of host-cell signaling pathways (26).

**Figure 2:**
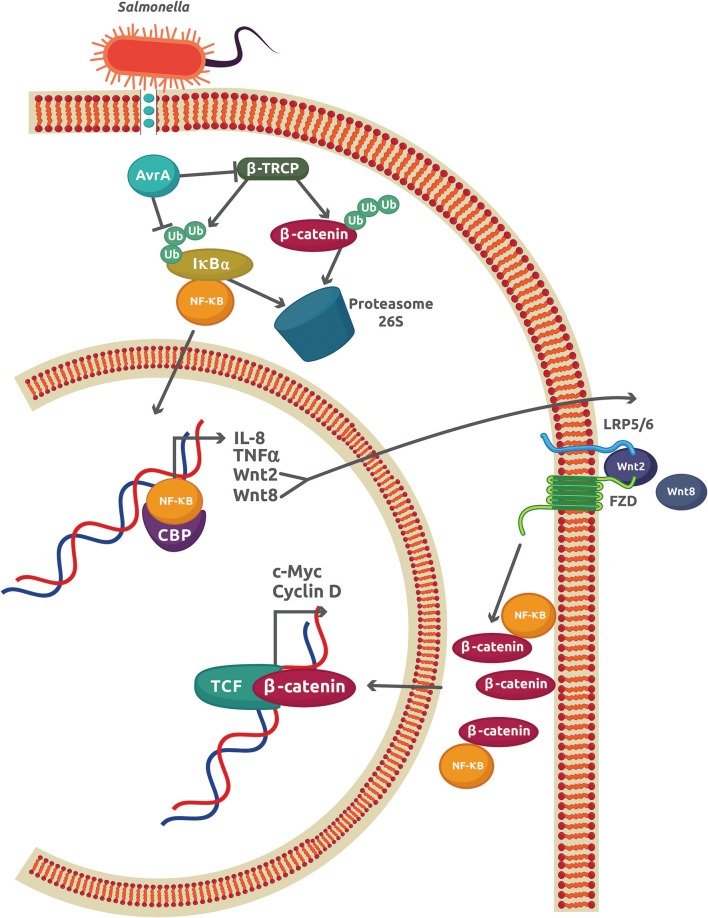
The AvrA virulence factor from *S. enterica* sv Typhimurium inhibits the inflammatory response in epithelial cells. The initial inflammatory response promoted by interaction of *S. enterica* with TLRs leads to an increase in the NF-κB activity and expression of the classical genes, IL-8 and TNFα, and also to Wnt2 and Wnt8. The autocrine effect of Wnt2/8 is to promote the cytoplasmic accumulation of β-catenin. Part of this β-catenin reduces the amount of free NF-κB by forming a complex with the NF-κB p50 subunit. *S. enterica* injects the virulence factor Avra through a type 3 secretion system. AvrA is also able to both inhibit the activity of β-TRCP and directly deubiquitinate IκBα, which increases the stability of the IκBα-NF-κB cytoplasmic complex and reduces the free NF-κB.

The best characterized *S. enterica* effector is the anti-virulence factor A (AvrA), a protein related to *Yersinia* acetyltransferase effector YopJ and the *Xanthomonas campestris* pv *vesicatoria* protein AvrBsT (27). AvrA promotes the inhibition of NF-κB activity by enhancing β-catenin deubiquitination in epithelial cells colonized with the non-pathogenic strain *S. enterica* SL1344 PhoPc AvrA^−^ or genetically complemented wild-type AvrA^−^/AvrA^+^ (28, 29). Direct interaction between the NF-κB p50 subunit and β-catenin caused a reduction of NF-κB-DNA binding and transcriptional activity, which inhibits the IR in *S. enterica* infections (30) (Figure 2). Apart from its stabilizing effect on β-catenin, AvrA is also an inducer of several Wnt glycoproteins expression including Wnt2, Wnt3, Wnt6, Wnt9a, and Wnt11 (13). The increase in Wnt2 and Wnt11 causes important reductions in IL-8 expression and *S. enterica* invasion, respectively [as reviewed in (31)]. Wnt3, Wnt6, and Wnt9a, which are important ligands that modulate the activity of stem cell differentiation, were also upregulated in mouse intestinal epithelial cells (IECs) (13). Recently, another member of the Wnt family, Wnt1, was shown to be affected by *S. enterica* (32). Expression of Wnt1 was decreased by ubiquitination and degradation in the proteasome 26S. Because AvrA is a deubiquitinase, this experimental finding may indicate a different mechanism used by AvrA to reduce the Wnt1 protein levels. This Wnt1 expression reduction in tumors of colon mucosa from mice led to an increase in the pro-inflammatory cytokines GM-CSF (granulocyte macrophage–colony stimulating factor), IL-6, and IL-8, which may promote a colitis-associated tumorigenesis (32). Thus, AvrA exerts a regulatory role on the host IR by inhibiting the NF-κB-dependent pro-inflammatory gene expression, inducing the activation of stem cells, and increasing the expression of pro-inflammatory cytokines in intestinal tumors as a consequence of Wnt1 expression inhibition. These effects observed in *S. enterica* infections may contribute to chronic disease and cell proliferation, leading to tumor development and cancer.

The Janus kinase (JAK)-signal transducer and activator of transcription (STAT) regulates many fundamental biological processes including cell proliferation and differentiation, inflammation, and apoptosis (33). In homeostatic conditions, STAT3 maintains under control the inflammation caused by indigenous gut microbiota. In chronic inflammation, the continuous stimulation of IECs by STAT3-activating cytokines (i.e., IL-6 and IL-15) increases the rate of cell proliferation, generates tumorigenic IECs, and causes loss of gut inflammation control (33). The activation of JAK-STAT3 signaling has also been associated with inflammatory bowel disease and colon cancer (34). Interestingly, AvrA activates the expression of the STAT3 target genes *MMP7* (matrix metalloproteinase-7) and *SOCS3* (suppressor of cytokine signaling 3) in a colon cancer mouse model (35). It is tempting to speculate that chronic activation of STAT3 synergizes with β-catenin to promote uncontrolled IEC proliferation and tumorigenesis.

AvrA structural and biophysical data were obtained last year by Labriola et al. (36). Its crystal structure showed many similarities with two YopJ acetyltransferases HopZ (a suppressor of immunity in *Arabidopsis thaliana*) from *Pseudomonas syringae* (37) and Pop2 (an inducer of immunity and inhibitor of proteasome activity in *A. thaliana*) from *Ralstonia solanacearum* (38). The 2.4-Å crystal resolution of AvrA allowed the identification of the secondary arrangement of the regulatory as well-as the catalytic site, and the binding sites of hexakisphosphate and acetyl-CoA (36). More importantly, they characterized the role of an α-helix near the catalytic site and a Leu^140^ residue as a determinant of substrate specificity. Such AvrA structural details have opened new possibilities to develop inhibitory molecules that would help block the harmful effects of this *S. enterica* effector.

## *Helicobacter pylori*: a Risk for Colorectal and Gastric Cancer

*Helicobacter pylori* is a spiral-shaped bacterium that causes more than 90% of duodenal ulcers and ~80% of gastric ulcers and cancer (39), and is present in about 67% of the population worldwide (40). Chronic infection by *H. pylori* is a strong risk factor for gastric cancer development. The virulent strains of *H. pylori* (Type 1 strains) contain the genomic cytotoxin-associated gene A pathogenicity island (*cagA*PAI) that mediates the assembly of a syringe-like structure type IV secretion system (T4SS) (40). Injecting *cagA* through the T4SS initiates a complex series of, as yet, not well-defined interactions with host cell molecules that are thought to be correlated with gastric diseases and cancer. Phosphorylated CagA can interact with many intracellular proteins such as Abl kinase (Abelson murine leukemia viral oncogene homolog 1), Crk (a proto-oncogene adaptor protein), Csk (C-terminal Src kinase), Shp2 (a protein tyrosine phosphatase encoded by the gene *PTPN11*), and others that contain an SH-2 domain (41). These CagA-SH-2 domain interactions influence the cytoskeleton rearrangement and increase cell mobility and size. The interaction of non-phosphorylated CagA with beta-1-integrin, E-cadherin, c-Met (a protein tyrosine kinase), P120-catenin, and ZO-1 (zonula occludens-1), just to cite a few (41), triggers inflammation and alterations in mitogenic signals, and disrupts cell junctions' structure. These phosphorylation-independent CagA activities and other mechanisms not related to CagA are directly associated with the accumulation of β-catenin in nucleus and gastric cancer emergence and development.

**Figure 3:**
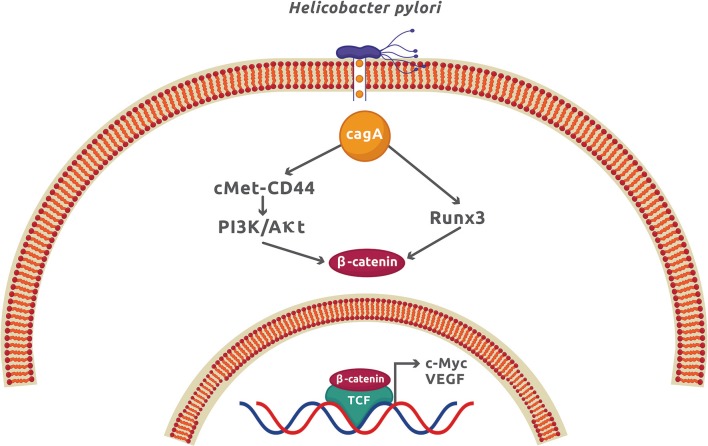
The CagA virulence factor from *Helicobacter pylori* promotes gastric cancer by activation of the Wnt/β-catenin signaling. Once CagA enters the cytoplasm by injection through the type 4 secretion system, it forms a molecular complex with c-Met and CD44, which activates the PI3K/Akt signaling. CagA also interacts with Runx3, which promotes its ubiquitination and proteasome degradation. These two CagA-dependent mechanisms, along with others independent of CagA described in the text, produces a net increment of cytoplasmic β-catenin and expression of β-catenin-dependent genes.

The infection by *H. pylori* causes colorectal and gastric cancer because it can activate the Wnt/β-catenin signaling pathway mainly by translocating the virulence factor CagA to the cytoplasm of epithelial cells. Once inside the host cell, CagA interacts and phosphorylates the oncoprotein c-Met receptor that, in turn, activates NF-κB and the expression of numerous pro-inflammatory cytokines and chemokines, enzymes, and angiogenic factors (41). The functional ternary complex CagA-c-Met-CD44 also induces nuclear β-catenin accumulation by activating the PI3K/Akt signaling (14, 42). This pathway inhibits the apoptotic cell death and is associated with colorectal cancer (43). The direct interaction of CagA with the gastric tumor suppressor transcription factor Runx3 (runt related transcription factor 3) labels it for ubiquitination and proteasome degradation (44). Runx3 is normally found interacting with Tcf4 and therefore repressing the β-catenin-dependent gene expression. The interaction of CagA with Runx3 exposes the Tcf4 binding site for β-catenin, which activates the upregulation of β-catenin target genes and induces stomach carcinogenesis (15) (Figure 3). Another tumor suppressor that is negatively affected by *H. pylori* is TFF1 (trefoil factor 1) (45). Akt inhibition by TFF1 increases the phosphorylating activity of GSK3β on β-catenin activating PP2A (protein phosphatase 2A). The net effect is the reduction of β-catenin nuclear translocation and Tcf4 transcriptional activity (45). In epithelial cells, *H. pylori* promotes the *TFF1* gene hypermethylation, leading to an increase in β-catenin-dependent gene expression (16). Promoter methylation and mRNA downregulation of Wnt/β-catenin antagonist genes secreted FRP (frizzled-related protein), DKK (DICKKOPF), and WIF1 (Wnt-inhibitory factor 1) have recently been demonstrated during gastric carcinogenesis and affected by *H. pylori* infection (17). As a result of this genetic modification, β-catenin nuclear translocation was increased in gastric epithelial cells. This study confirms that epigenetic modification is an important factor for gastric cancer, in which *H. pylori* plays a significant role. Apart from epigenetic modifications, *H. pylori* infection promotes cancer stem cell characteristics in cancer gastric cells by activating Wnt/β-catenin signaling in a process dependent on CagA (46). It was also found that Nanog and Oct4, two transcription factors associated with epithelial–mesenchymal transition (EMT), increased their expression in the gastric cancer samples from patients infected with *cagA*-positive *H. pylori* (47).

*Helicobacter pylori* can induce gastric cancer by activating cell proliferation, cell invasion, and angiogenesis (48). The activation of Wnt/β-catenin signaling is mainly responsible for cell proliferation in gastric tumorigenesis (49). Interestingly, a new mechanism to activate Wnt signaling was recently discovered by Geng et al. (18). These authors showed that *H. pylori* upregulates the Wnt receptor Fzd7 and simultaneously represses miR-27b. The inactivation of miR-27b, which has been identified as a tumor suppressor (50, 51), resulted in the overexpression of Fzd7 followed by Wnt signaling activation. Moreover, Wnt/β-catenin activation in *H. pylori* infection has been linked to angiogenesis in the gastric mucosa, which is an important process for tumorigenesis and development of gastric cancer (52). This mechanism involves the upregulation of cyclooxygenase 2 (COX-2) and inhibition of β-catenin phosphorylation. As a result, β-catenin accumulates in the cytoplasm and then translocates to the nucleus. Finally, COX-2/Wnt/β-catenin signaling activation leads to an increase in vascular endothelial growth factor (VEGF) expression, a strong proangiogenic molecule (52).

## *Mycobacterium tuberculosis*: A Wnt–Fzd Interaction That Defines the Infection Outcome

**Figure 4:**
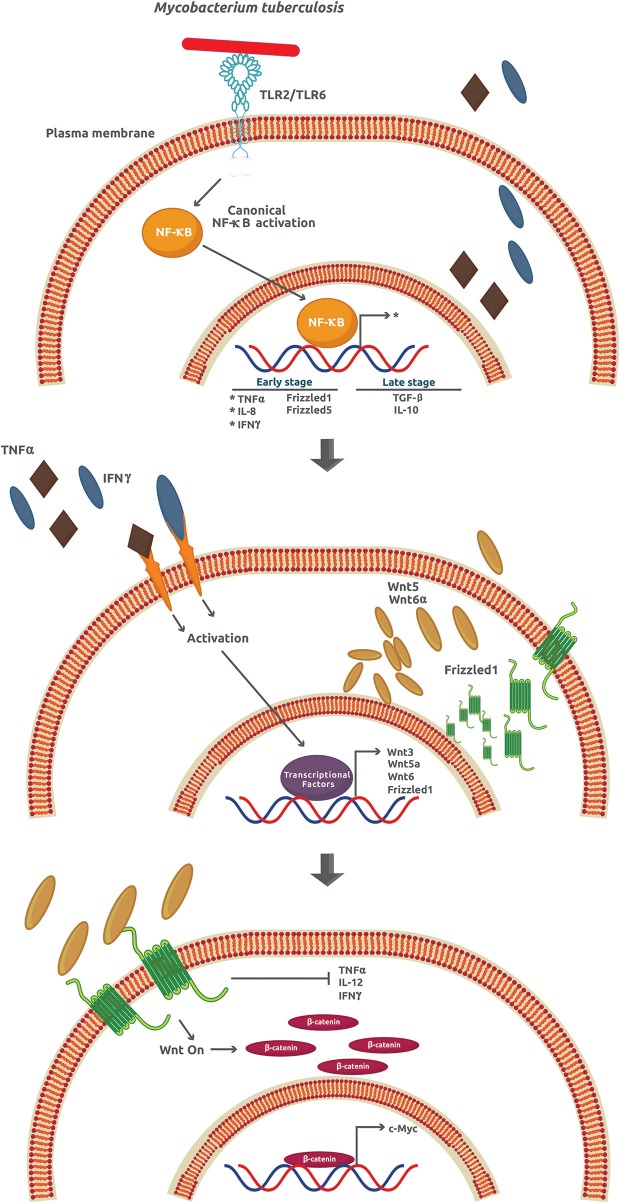
*Mycobacterium tuberculosis* activates several signaling pathways that lead to an inflammatory response inhibition. During the early phase of infection, *M. tuberculosis* induces the expression of TNFα, IL-6, IFNγ, Frizzled1, and Frizzled5. However, at later stages of infection, there is an increase of the anti-inflammatory cytokines TGFβ and IL-10. The secreted TNFα and IFNγ promote the paracrine expression of Wnt3, Wnt5a, Wnt6, and Frizzled1 that in turn activates the Wnt/β-catenin signaling.

Tuberculosis (TB), the name of the infection caused by *M. tuberculosis* in humans, is one of the most widespread and deadly diseases worldwide with approximately 25% of the whole population infected. The number of TB cases in 2017 around the world was about 10 million, with 1.3 million TB-associated HIV patient deaths (53). Once *M. tuberculosis* invades the lungs, it infects the alveolar macrophages in a process associated with, but not strictly dependent on, several membrane receptors including C-type lectin receptors, mannose receptors, scavenger receptors, CD14, CD43, and LSPA (lung surfactant protein A) [as reviewed in (19)]. The success of *M. tuberculosis* as an intracellular pathogen depends on its ability to manipulate many signaling mechanisms activated by the host cell. For example, after infection, a burst of pro-inflammatory cytokines and chemokines like TNFα (tumor necrosis factor α), IL-1β, and IL-6 is observed followed by the expression of anti-inflammatory cytokines such as TGF-β (transforming growth factor-β) and IL-10 [as reviewed in (54)]. It is assumed that the attenuation of the inflammatory and adaptive response through production of IL-10 is critical for *M. tuberculosis* survival and granuloma formation (Figure 4, upper panel).

Wnt glycoproteins play a functional role as pro- or anti-inflammatory factors depending on the cellular type and context, the stimuli, and the kind of cytokines previously secreted (1). In particular, when cells are infected by *M. tuberculosis*, Wnt3a and Wnt5a downregulation and Wnt6 upregulation promote an anti-IR, *via* canonical (Wnt3a) and non-canonical (Wnt5a and Wnt6) signaling in mouse and human models (19). In contrast to Wnt3 that prevents infection by *M. tuberculosis*, Wnt5a and Wnt6 promote it. Except for Wnt5a, Wnt3, and Wnt6, expression is not directly induced by *M. tuberculosis* (Figure 4, upper and middle panel). However, the upregulation of Fzd1 and Fzd5 is a process directly related to the effect of *M. tuberculosis* (19). The downregulation of Fzd3–4 and Fzd6–10 has been correlated with reduced β-catenin-dependent gene expression (55). Apart from the effects on inflammation, Wnt3, Wnt5a, and Wnt6 inhibit necrosis, TNFα secretion, IL-12 and IFNγ production, phagolysosome formation, promotion of apoptosis and phagocytosis, cellular adhesion, and cellular proliferation, among others (Figure 4, lower panel). Collectively, these effects help *M. tuberculosis* survive and damage the intracellular milieu and surrounding cells (19).

## *Pseudomonas aeruginosa*: Targeting Wnt/β-Catenin to Promote Bacterial Survival

*P. aeruginosa* is an opportunistic bacterial pathogen associated with severe intra-hospital pneumonia (56). One of its most common pathogenic mechanisms leading to the recurrence and chronic infections is to damage the epithelial monolayer structure in the lung alveoli. Also, *P. aeruginosa* avoids elimination by macrophages, which promotes its prevalence and produces chronic inflammation (57).

Experiments with murine macrophages RAW264.7 infected with *P. aeruginosa* 19660 have demonstrated a time-dependent increase in β-catenin degradation (58). In contrast, the overexpression of the β-catenin gene *CTNNB1* in macrophages enhances bacterial intracellular killing by counteracting cell auto-phagocytosis without affecting reactive oxygen species synthesis. However, *P. aeruginosa* can survive inside macrophages by promoting β-catenin degradation (58). These experimental observations indicate that, in macrophages, β-catenin promotes an auto-phagocytosis-breakresistant phenotype.

In an acute lung injury model, *P. aeruginosa* infection of epithelial H1299 and H1975 cell lines promotes NF-κB activation by stimulating its nuclear translocation and β-catenin degradation (11). Interestingly, this degradation was linked to the virulence factor LecB because incubating H1299 cells with recombinant purified LecB was sufficient to induce β-catenin degradation and repress the expression of the β-catenin-dependent genes *c-Myc* and *Cyclin-D1*. Thus, LecB causes a reduction in cellular proliferation by arresting cell cycle in G1 to S phase transition. LecB also promoted β-catenin intracellular reorganization by destabilizing its molecular complex with α1β3 integrin in the plasma membrane and β-catenin degradation in a process dependent on GSK3α/β but independent on PI3K/Akt (11). In agreement with these data, Wnt3a, a prototypical regulator of β-catenin destruction complex, prevented LecB-induced β-catenin degradation (11). These results suggest a role for β-catenin in modulating cellular functions directly related to *P. aeruginosa* clearance.

## *Citrobacter rodentium*: an Unknown Bacterial Effector Promoting Hyperplasia

This bacterium is an enteric pathogen of mice that shares mechanistic similarities with the classical enteropathogenic and enterohemorrhagic strains of *Escherichia coli* (EPEC and EHEC). This feature constitutes a useful model to investigate the characteristic attaching/effacing (A/E) injuries of the gut epithelium induced by *E. coli* in humans (59). Apart from the A/E lesions, *C. rodentium* induces hyperplasia and tumorigenesis of the crypt cells (60). A detailed analysis has revealed that mesenchymal stem cells are the primary target of hyperplasia and the activation of Wnt/β-catenin signaling has been correlated to the self-renewal phenotype and proliferation (60). Wnt/β-catenin signaling also promotes an increase in R-spondin-2 expression, an agonist of the leucine-rich repeat G-protein coupled receptor (Lgr), that, along with Fzd and LRP5/6, regulates the expression of Mmp7 and c-Myc genes involved in cell regeneration (9). Although a specific bacterial effector responsible for these mechanisms has not been identified, the hyperplasia caused by *C. rodentium* infection requires an intact T3SS to promote cellular proliferation and Wnt/β-catenin activation (60). Infecting mice with the T3SS defective mutant ΔEscV (an inner membrane T3SS assembly related protein) fails to induce the expression of proliferation markers and attenuates hyperplasia (10, 61).

*C. rodentium* infection also promotes Wnt/β-catenin-mediated hyperplasia through the repression of the Wnt/β-catenin inhibitor factor 1 (WIF1) expression in two different ways. The first mechanism involves epigenetic modification of the enhancer region of the methyltransferase zeste homolog-2 (*EZH2*) gene that, in turn, promotes the H3 lysine-27-trimethylation (H3K27me3) in the promoter region of the WIF1 gene (10). The second mechanism involves the downregulation of WIF1 gene by the upregulation of miRNA203. Interestingly, these mechanisms are dependent on bacterial effectors because the infection of mouse colonic primary cells with the *C. rodentium* mutants ΔEscV showed a reduced expression of *EZH2* and *H3K27me3* genes and an increased expression of *WIF1* compared with their non-mutant counterparts (10). So far, it is uncertain why *C. rodentium* promotes stem cell hyperplasia. It is likely that elevated levels of β-catenin may block the NF-κB pathway (62), which is one of the main defense mechanisms against bacterial infections.

## *Clostridium difficile*: Hijacking Wnt–Fzd Interaction

This bacterium is a human commensal and opportunistic pathogen related to pseudo-membranous colitis and antibiotic-associated diarrhea, which can be life-threatening diseases among elderly patients in developed countries. The most virulent strains secrete toxins A and B. These toxins bind to chondroitin sulfate proteoglycan 4 (CSP4) in HeLa cells and inhibit small GTPases by glycosylation resulting in cell rounding and death (63, 64); however, CSP4 was not detected in colonic epithelium where *C. difficile* inhabits, indicating the existence of additional receptors involved. The evidence obtained by genetic screening analysis suggests that the Wnt receptors Fzd1, 2, and 7 from HeLa, Caco-2, and HT-29 cells interact with toxin B (7, 65). When toxin B binds to Fzd receptors, Wnt3a and probably other Wnt ligands cannot activate the Wnt/β-catenin pathway (7, 66). The inhibition of Wnt/β-catenin signaling by toxin B from *C. difficile* represses the TCF-dependent gene expression.

Toxin A, on the other hand, can attenuate Wnt/β-catenin signaling by repressing target genes expression like *c-Myc* even in the presence of the GSK3 inhibitor LiCl, indicating the existence of additional steps in its inhibitory mechanism (8). A precise role for Wnt/β-catenin pathway inhibition during *C. difficile* infections remains unclear. Further research on these pathogenic mechanisms is strongly recommended because this bacterium is difficult to control and may cause fatal diseases (63).

## *Bacteroides fragilis*: Targeting β-Catenin From the Adherens Junctions

This bacterium constitutes around 1 to 2% of the gut microbiota and approximately 30% of the anaerobic organisms cultured from human feces. It is considered as the most frequent anaerobic pathogen capable of infecting soft tissues and an important etiological agent of diarrhea (67). The genome of *B. fragilis* virulent strains contains a pathogenic island that encodes a *B. fragilis* toxin (BFT), which is a heat-labile metalloprotease. Rabbits challenged with high concentrations of BFT show tissue damage and hemorrhage, an increase in IL-8 expression, NF-κB activation, and disruption of the epithelial barrier (68, 69).

BFT was the first bacterial effector reported to activate the β-catenin-dependent gene expression (12). The protease-mediated activity of BFT on the extracellular domain of E-cadherin has been suggested as the mechanism to disrupt epithelial cell-to-cell contact. As a result, BFT causes the dissociation of β-catenin from E-cadherin and, once released, β-catenin translocates to the nucleus where it forms a complex with TCF4, leading to *c-Myc* expression and cellular proliferation in the APC mutant cell lines HT29/C1 and SW480 (12). β-Catenin released from E-cadherin interaction do not translocate directly to the nucleus but to the perinuclear endocytic recycling compartment in A431 cells that express full APC protein (70). This observation indicates that the impaired function of the β-catenin *destruction complex* is a condition needed for BFT to induce β-catenin-mediated effects. However, the effects induced by *B. fragilis* infection on the β-catenin *destruction complex* proteins in non-APC mutant cells are still unknown and deserve further investigation. In agreement with this reasoning, *Bordetella pertussis* toxin, which has been reported to induce the dissociation of β-catenin from the complex with VE-cadherin of adherens junctions, also promotes GSK3-specific phosphorylation at Thr41 and Ser45 of the released β-catenin that marks it for degradation (71).

## *E. coli*: Osteogenic Differentiation of Human Periodontal Stem Cells by LPS

Periodontitis is an inflammatory disease caused by microbial populations in oral microbiota, which implies biofilm formation and deregulation of the IR from the host (72). Gram-negative anaerobic bacteria are among the most prominent bacterial species found in periodontal disease. Lipopolysaccharide (LPS) is a common cell surface antigen present in Gram-negative bacteria. Recently, Xing et al. (20) found that LPS from *E. coli* stimulated osteogenic differentiation of human periodontal ligament stem cells (HPDLSCs) but not their proliferation, viability, or cell cycle regulation. They showed that this stimulation was driven by the activation of Wnt/β-catenin pathway. Osteogenesis and osteogenesis stimulated by LPS were confirmed by RT-PCR of osteogenesis-related genes ALP (alkaline phosphatase), RUNX2 (Runt-related transcription factor 2), OCN (osteocalcina), and OSX (osterix) (20). TAZ (also known as WWTR1 [domain containing transcription regulator 1]) is a key transcriptional co-activator with a PDZ binding motif that mediates cell proliferation, differentiation, and stem cell renewal through the Wnt/β-catenin. Its mRNA and protein levels were also increased during LPS-stimulated osteogenesis. The depletion of TAZ by an antiviral transfection with a shTAZ construction reduced both LPS-stimulated and independent osteogenesis *in vitro* and also blocked the induction of osteogenesis-related genes, emphasizing its major role in osteogenesis (20). To demonstrate the role of Wnt/β-catenin in the induction of TAZ and osteogenesis, LPS stimulation was performed in the presence of DKK1 (DIKKOPF1), a known inhibitor of the Wnt/β-catenin transduction pathway that blocks the interaction between Fzd and LRP5/6 with Wnt ligands. ALP or alizarin red staining assays showed that DKK1 inhibited osteogenesis *in vitro*, in the presence or absence of LPS. This was accompanied by a decrease in the accumulation of TAZ and β-catenin in the presence of LPS, and a slight but significant increase in phosphorylated TAZ at Ser89. DKK1 also reversed the induction of osteogenesis related-genes by LPS (20).

## *Haemophilus parasuis*: Initiation of EMT

Glässer's disease (Gd) is an acute infection in pigs characterized by a combination of meningoencephalitis, polyserositis, pericarditis, polyarthritis, and, in some cases, primary pneumonia. It is caused by specific serovars of *H. parasuis*, a bacterium that normally resides in the upper respiratory tract. In certain host stress conditions (i.e., weaning, antibiotic treatment, and transportation), *H. parasuis* may turn pathogenic, causing Gd or an acute respiratory disease, depending on the site of infection. Sometimes, an acute inflammatory systemic response causes massive fibrin exudates in the pleuroperitoneal cavity leading to sudden death (73).

The disruption of adherens junctions is a major alteration of epithelial cells that leads to the modification of epithelial permeability. Recently, a role for Wnt/β-catenin in the EMT that contributes to the loss of epithelial permeability caused by *H. parasuis* was reported (21). The virulent *H. parasuis* strain SH0165 was observed to increase β-catenin and reduce phospho-β-catenin at Ser33/37/Thr41 as compared with the non-virulent strain HN0001 in pig kidney (PK-15) cell line and newborn pig tracheal (NPTr) epithelial cell lines. Relative luciferase expression assays showed that the Wnt/β-catenin pathway was activated by increasing doses of the virulent strain and by LiCl, a known GSK3β inhibitor that stimulates the Wnt signaling pathway. This activation correlated well with an increase in β-catenin and a decrease in phospho-β-catenin. β-Catenin was also translocated from the membrane to the cytoplasm and to the nucleus by inoculation with the virulent strain and treatment with LiCl (21). E-cadherin was also degraded upon the interaction of epithelial cells with the virulent strain or by stimulation with LiCl; this was reversed by adding Wnt/β-catenin inhibitors ICG001 and IWR-endo-1, demonstrating the role of the signaling pathway in maintaining the stability of adherens junctions (21). Furthermore, nuclear immunolocalization of β-catenin and reduction of E-cadherin levels in the membrane were confirmed by confocal laser scanning microscopy (CLSM), and these correlated with alterations in epithelial monolayer permeability. The final demonstration of Wnt signaling activity in EMT was conducted by applying siRNA technology against several Wnt target genes such as *MMP7* (membrane palmitoylated protein 7), *PAI-1* (plasminogen activated inhibitor 1), and *COX2* (cyclooxygenase 2), which significantly produced a recovery of the expression levels of the EMT-related genes *E-cadherin, collagen IV, cytokeratin, N-cadherin, Snail, Vimentin*, and *S100A4* (21).

## *Lawsonia intracellularis*: Alteration of Homeostasis and Promotion of Cell Proliferation During Infection of Intestinal Crypt Cells

Proliferative entheropathy (PE) is an infectious disease caused by *L. intracellularis* in which bacteria invade the intestinal crypt cells inducing extensive cell proliferation and loss of the integrity of intestinal mucosa and mucosal thickening. The disease may have two forms—an acute one characterized by hemorrhagic diarrhea and sudden death, and a chronic one with no hemorrhagic diarrhea that causes wasting and loss of control (74).

Wnt/β-catenin and Notch signaling regulate intestinal stem cell (ISC) proliferation and differentiation. These pathways transit amplifying (TA) progenitor cells from which secretory cells and intestinal enterocytes differentiate, regulating in that way the intestinal homeostasis. The evaluation of Wnt/β-catenin and Notch signaling during the disruption of intestinal homeostasis caused by *L. intracellularis* infection was investigated by Huan et al. (22). They detected fluorescent *L. intracellularis* in the crypts of ileum sections and observed an increase in bacterial number from 3 to 14 days post-colonization (dpc) and a drastic decrease by 28 dpc. Fluorescence and mRNA levels of MUC2 (intestinal mucin 2) reached the lowest levels by 14 dpc, consistent with the maximum of *L. intracellularis* fluorescent signal in the ileum. A cell proliferation marker Ki67 and the apoptosis marker caspase-3 also reached their highest fluorescent signals by 14 dpc. These assays demonstrated the correlation between *L. intracellularis* ileum crypt colonization and the increase in cell proliferation and apoptosis, which result in PE tissue damage. β-Catenin also increased in the upper and lower halves of the crypt by 14 dpc, which was in agreement with the repression of the Wnt/β-catenin-dependent genes *ASCL2* (achaete-scute family BHLH transcription factor 2), *LGR5* (leucine-rich repeat containing G-protein receptor 5), *SOX9* (SRY box 9), and *Cyclin D1* (22). The intercellular Notch-1 receptor domain/C-terminal (NICD1) immunodetection in crypts also reached its highest level at 14 dpc, correlating with the transcript repression of Notch-regulated genes *ATOH1* (atonal BHLB transcription factor 1; 14 dpc), *HES1* (hairy and enhancer ff split1; 21 dpc), and *OLFM4* (olfactomedin 4; 28 dpc) (22). Changes in Wnt/β-catenin- and Notch-regulated gene expression were consistent with previous reports on their role in intestinal homeostasis, allowing authors to suggest the participation of these major signaling pathways in PE lesions.

## *Shigella dysenteriae*: IR Through Wnt/β-Catenin and NF-κB Pathways

*Shigella dysenteriae* is one of the four species of the genus. It is integrated by 15 serotypes, which are one of the main causative agents of dysentery in developing countries. It destroys the colonic epithelium without causing systemic infection. The typical symptoms produced are diarrhea with blood and mucus accompanied by abdominal cramps and fever (75). Colon infection is associated with an acute IR mediated by IL-8. Recently, Gopal et al. (23) explored the involvement of the Wnt/β-catenin and NF-κB pathways in regulating IL-8 levels and the IR. A rat ileal loop infection model was used to test the IR and the signaling pathways involved. The infection of the ileal loop with *S. dysenteriae* increased both the protein and mRNA expression levels of proinflammatory cytokines IL-8 and TNFα. Immunohistochemical detection reveals that both β-catenin and NF-κB transcription factors increased the level of expression and its localization (from cytoplasmic to nuclear) after infection with *S. dysenteriae* (23). Phospho-IκBα, NF-κB, phospho-β-catenin, and GSK3β phosphorylated at Y216, and proteins showed increased levels after infection, whereas unphosphorylated β-catenin and phospho-GSK3β at S9 were reduced. Co-immunoprecipitation with GSK3β and β-catenin antibodies demonstrated the association of β-catenin and NF-κB through GSK3β. Overall, these data suggest that *S. dysenteriae* negatively regulates β-catenin pathway by modulating GSK3β activity; also, the formation of a β-catenin/GSK3β complex promotes β-catenin degradation and release of NF-κB from the ternary complex, leading to inflammation (23).

## *Staphylococcus epidermidis*: Inhibition of Skin Inflammation by β-Catenin Activation

*Staphylococcus epidermidis* protects the skin from pathogen infections by secreting antimicrobial peptides (76), although, in some specific conditions, it may become an opportunistic pathogen. Lactic acid bacteria have also been associated with skin wound healing because they can produce bioactive metabolites that control pathogen bacteria and modulate the immune response (77). The ability of commensal *S. epidermidis* to contribute to wound healing was recently examined by Li et al. (24). These authors isolated lipopeptide 78 (LP78; a peptide of 23 amino acids with an heneicosanoic acid bound to the N-terminal aspartic acid D1) from culture medium and tested its effects on IR in a neonatal human epidermal keratinocytes (NHEKs) cell line and in streptozotocin-induced type 1 diabetic mice. When NHEKs were pre-treated with poly(I:C) and different LP78 concentrations, a significant reduction of TNFα and IL-6 was observed (24). The inhibitory effect of LP78 was potentiated by the addition of lipoteichoic acid (LTA), one of the main proinflammatory structures present in the cell wall of Gram-positive bacteria.

When TNFα and IL-6 protein levels were estimated in the wounds of normal mice or in the wounds of *Tlr-3*^−/−^ deficient mice, LP78 repression of cytokine expression was observed in normal but not in TLR3-deficient mice (24). Immunolocalization by CLSM and quantification of the NF-κB p65 subunit suggested that LP78 does not block NF-κB nuclear translocation induced by poly(I:C). In NEHKs, LP78 induced accumulation of phospho-β-catenin at Y654 and translocation of β-catenin to nucleus in a time-dependent manner. The LP78-mediated repression of poly(I:C)-induced cytokines is lost in NEHK cells with shRNA-reduced levels of β-catenin and in wounds treated with the β-catenin inhibitor FH535, which also caused a decrease in LP78-induced phospho-β-catenin (Y654) accumulation (24). The TLR2 inhibitor OxPAPC (oxidized 1-palmitoyl-2-arachidonyl-sn-glycero-3-phosphorylcholine) caused a reduction in β-catenin nuclear translocation and LP78 repression of poly(I:C)-induced cytokines. LP78 effect is also lost in a TLR2-deficient genetic background in the ear intradermic injection model and in primary culture of murine keratinocytes and in the skin wound model. These data suggest that LP78 activates TLR2. To elucidate the TLR2 pathway, inhibitors such as PP2 (specific for non-receptor tyrosine kinase family Src), Bay11 (specific for NF-κB), SB431542 (specific for TGF-β), and FH535 (specific for β-catenin) were tested (24). PP2 and FH535 blocked LP78-induced β-catenin phosphorylation. Therefore, these data suggest that LP78 activates TLR2-Src to induce β-catenin activation. GW9662, an inhibitor of PPARγ (peroxisome proliferator-activated receptor gamma), inhibited poly(I:C)-induced TNFα and IL-6. As it is known that PPARγ interacts with p65 and that this interaction is disrupted by β-catenin, experiments were conducted to elucidate if LP78 inhibits TLR3-mediated IR by β-catenin-mediated impairment of p65–PPARγ interaction (24). NEHK cells treated with poly(I:C) and stimulated with LP78 showed reduced amounts of p65. Under these conditions, β-catenin amounts decreased in the cytoplasm and increased in the nucleus. Altogether, these data suggested that LP78 induces β-catenin nuclear translocation. Once in the nucleus, β-catenin disrupts the interaction between TLR3-activated p65 and PPARγ, thus inhibiting TLR3-mediated inflammation in skin wounds.

## Concluding Remarks

The *golden age* of antibiotics therapy against the most important human pathogenic bacteria is approaching a dead end. Nosocomial infections caused by several bacteria cited in this review are difficult to eliminate because of multiple mechanisms that make them resistant to antibiotics specifically recommended for their treatment (78). This antibiotic resistance has generated the appearance of *superbugs*, which are responsible for epidemics with enhanced morbidity and mortality.

One of the most promising alternatives to overcome the bacterial resistance to antibiotics is to search for the host cell mechanisms that are activated or inhibited by virulence factors secreted during pathogenic bacteria infections and then identify the protein molecules involved. Protein kinases (i.e., p38, JNK) and specific transcription factors (i.e., NF-κB), as well as cytoskeletal targets activated during the pro-IR, have been characterized in a number of bacterial infections. More recently, signaling pathways like Wnt/β-catenin and Notch, whose main function is not related to inflammation, were added to the arsenal of molecular mechanisms touched by pathogenic bacteria. The complexity is even greater because the classical NF-κB pro-inflammatory signaling molecules cross-talk with other signaling pathways such as the Wnt/β-catenin, which are not apparently related to inflammation. This interrelationship frequently gives unexpected phenotypes that need to be taken into account to understand the temporal stages of infection. An integration of signaling at each stage and its proper interpretation may help researchers identify the important clues to fight lethal bacteria. Once signaling molecules are identified, the next step would be to design specific drugs to deactivate the virulence factors. In order to accomplish this ambitious goal, the structural details of virulence factors need to be elucidated. Such biochemical studies combined with bioinformatics, molecular dynamics approaches, and traditional methods (i.e., vaccines) will undoubtedly pave the way to win the battle against infectious diseases.

## Author Contributions

OS-G and JV-A helped with the writing of the manuscript. VB-A conceived, wrote, edited, and reviewed the manuscript.

### Conflict of Interest Statement

The authors declare that the research was conducted in the absence of any commercial or financial relationships that could be construed as a potential conflict of interest.
